# Several Important *In Vitro* Improvements in the Amplification, Differentiation and Tracing of Fetal Liver Stem/Progenitor Cells

**DOI:** 10.1371/journal.pone.0047346

**Published:** 2012-10-09

**Authors:** Wei-hui Liu, Zheng-cai Liu, Nan You, Ning Zhang, Tao Wang, Zhen-bin Gong, Hong-bao Liu, Ke-feng Dou

**Affiliations:** 1 PLA Center of General Surgery, General Hospital of Chengdu Army Region, Chengdu, Sichuan Province, People's Republic of China; 2 Department of Hepatobiliary Surgery, Xijing Hospital, Fourth Military Medical University, Xi'an, Shaanxi Province, People's Republic of China; 3 Department of Nephrology, Xijing Hospital, Fourth Military Medical University, Xi'an, Shaanxi Province, People's Republic of China; Instituto Butantan, Brazil

## Abstract

**Objective:**

We previously isolated fetal liver stem/progenitor cells (FLSPCs), but there is an urgent need to properly amplify FLSPCs, effectively induce FLSPCs differentiation, and steadily trace FLSPCs for *in vivo* therapeutic investigation.

**Methods:**

FLSPCs were maintained *in vitro* as adherent culture or soft agar culture for large-scale amplification. To direct the differentiation of FLSPCs into hepatocytes, FLSPCs were randomly divided into four groups: control, 1% DMSO-treated, 20 ng/ml HGF-treated and 1% DMSO+20 ng/ml HGF-treated. To trace FLSPCs, the GFP gene was introduced into FLSPCs by liposome-mediated transfection.

**Results:**

For amplifying FLSPCs, the soft agar culture were more suitable than the adherent culture, because the soft agar culture obtained more homogeneous cells. These cells were with high nuclear:cytoplasmic ratio, few cell organelles, high expression of CD90.1 and CD49f, and strong alkaline phosphatase staining. For inducing FLSPCs differentiation, treatment with HGF+DMSO was most effective (*P*<0.05), which was strongly supported by the typical morphological change and the significant decrease of OV-6 positive cells (*P*<0.01). In addition, the time of indocyanine green elimination, the percentage of glycogen synthetic cells, and the expressions of ALB, G-6-P, CK-8, CK-18 and CYP450-3A1 in HGF+DMSO-treated group were higher than in any other group. For tracing FLSPCs, after the selection of stable FLSPC transfectants, GFP expression continued over successive generations.

**Conclusions:**

FLSPCs can properly self-renew in soft agar culture and effectively differentiate into hepatocyte-like cells by HGF+DMSO induction, and they can be reliably traced by GFP expression.

## Introduction

The current strategies to restore liver mass and functionality are aimed at either the transplantation of hepatocyte-like cells or the stimulation of endogenous liver repair [1]. The transplantation of hepatocytes has been evaluated in clinical experiments. Its long-term efficacy remains unclear, and the paucity of donor cells limits this strategy. Stem cell transplantation is a more promising approach [2]. Among the stem/progenitor cells in the treatment of liver diseases [3]–[12], fetal liver stem/progenitor cells (FLSPCs) are speculated to have the most therapeutic potential because they can repopulate hepatocytes and bile ducts [9], [13], [14].

With a novel and simple method [a combination of Percoll discontinuous-gradient centrifugation (PDGC), differential trypsinization and differential adherence (DTDA) and Percoll continuous-gradient centrifugation (PCGC)], we have successfully isolated FLSPCs from fetal rat livers [15]. Especially, the efficiency of our novel method is close to that of magnetic affinity cells sorting (MACS). Briefly, the fetal liver cells (FLCs) were firstly enriched by PDGC from the rat fetal liver of embryonic day 14. Because FLCs contained relatively miscellaneous cell types, in culture they were purified to be homogeneous in size by DTDA. At last, FLCs were layered into six different cell populations by PCGC according to cell densities. Because FLSCPs were thought to be the smallest cells in FLCs, the cells located in the first upper layer of PCGC gradient were isolated and identified as FLSPCs. The stem characterization of FLSPCs included three aspects: detecting the expression of stem cell markers, investigating the self-renewal ability and multiple differentiation potential. Firstly, FLPSCs should highly express stem cell markers, such as CD133 and CD49f. Secondly, FLSPCs could rapidly duplicate themselves *in vitro* culture. Thirdly, after induction by hepatocyte growth factor (HGF), FLSPCs could generate albumin (ALB) positive hepatocytes and cytokeratin 7 (CK-7) positive bile duct cells. To conclude, because the isolated cells by our method achieved all the above standards, they could be identified as FLSPCs [15].

Although FLSPCs are successfully isolated, before they can be applied to treating liver diseases even in animal models, several technical obstacles must be overcome. For example, the present conditions for amplifying FLSPCs are not satisfying, and the effective strategy for inducing FLSPCs differentiation is scanty. To conquer the above difficulties, in this study we made several *in vitro* important improvements to push FLSPCs into therapeutic investigation. First of all, we engaged in finding a proper way to amplify FLSPCs in large-scale, so as it could provide quantity guarantee for treating liver diseases. Inspired by investigations focusing on neural stem cells (NSCs) culture [16], we found that soft agar culture was well suited to amplify and maintain FLSPCs. Although FLSPCs can be largely amplified by soft agar culture, because they are immature and lack function, they have to differentiate into hepatocytes to restore normal liver functioning. Therefore, in this study by combining the hepatic differentiation inducers HGF [17]–[19] with dimethyl sulfoxide (DMSO) [20], [21], we successfully induced the differentiation of FLSPCs into hepatocyte-like cells. Thus, this effective strategy could provide quality assurance for treating liver diseases. When FLSPCs are prepared for *in vivo* transplantation, they should be well traced so that we can make sure whether FLSPCs can implant into damaged liver and generate hepatocytes to repair the injured liver. To achieve the above goal, in this study we linked FLSPCs with a steady tracer. Green fluorescent protein (GFP) is a widely used cell tracer [22]–[25]. When cells are labeled with GFP, their proliferation and differentiation characteristics can be monitored both *in vivo* and *in vitro*.

In one word, based on the present research progress, in this study we made three important *in vitro* improvements in pushing FLSPCs for treating liver diseases. The insights gained from this study will be helpful in designing the optimal protocols for FLSPC-based cell therapy.

## Materials and Methods

### 1 The amplification of FLSPCs

#### 1.1 In vitro culture of FLSPCs

The FLSPCs were freshly isolated from fetal rat livers at embryonic day 14 according to our previous method [15]. The FLSPCs were randomly divided into two groups: control group and experimental group. The FLSPCs in the control group were seeded onto type I collagen-coated plates (5000 cells/cm^2^) for adherent culture. The medium in the control group was 1× Williams' Medium E containing 15% FBS, 20 µg/ml epidermal growth factor (EGF) and 10 µg/ml leukemia inhibitory factor (LIF). The FLSPCs in the experimental group were seeded between two soft afar layers for soft agar culture (sandwich culture). The medium in the basal layer was composed of equal volumes of 1.2% agar and 2× Williams' Medium E containing 40% FBS and 20 µg/ml EGF (Applichem, Darmstadt, Germany). The upper layer was composed of equal volumes of 0.6% agar and 1× Williams' Medium E containing 10% FBS and 10 µg/ml LIF. All of the cells were maintained at 37°C in a humidified atmosphere containing 5% CO_2_ for 2 weeks.

#### 1.2 The morphological comparison of the FLSPCs in two groups

Over the time of the culture, the morphological characteristics of the FLSPCs in both groups were observed and captured with a CKX-41 inverted microscope (Olympus Corporation, Tokyo, Japan) and recorded on days 1, 7, 14, 21 and 35. After 14 days of culturing, the FLSPC samples from both groups were collected and washed twice with phosphate-buffered saline (PBS) to prepare the cells for sectioning and observation with a JEM-1200EX transmission electron microscope (TEM) (JEOL Company, Tokyo, Japan). The protocol for sectioning was as follows: the cells were fixed sequentially with 2.5% glutaraldehyde and 1% osmium tetroxide, dehydrated continuously with ethanol and acetone, infused with epoxide resin, embedded in paraffin, sliced into ultrathin sections, and stained by uranium acetate and lead citrate.

#### 1.3 Alkaline phosphatase (ALP) staining of the FLSPCs in two groups

The identification of ALP-positive clones is the gold standard by which undifferentiated stem cells are evaluated [26]–[29]. After culture for 14 days, samples from both groups were fixed with 4% paraformaldehyde and stained with ALP using the Alkaline Phosphatase Detection Kit (Millipore Corporation, Massachusetts, USA). The positive cells were stained red as observed in an inverted microscope. To quantify the percentage of ALP-positive cells in the experimental group, cell spheres were sequentially digested by trypsin (a short time, approximately 5 min) and scattered by repeated pipetting to prepare them as single-cell suspensions. These single-cell suspensions were viewed under an inverted microscope and the positive cells were calculated.

#### 1.4 The functional comparison of the FLSPCs in two groups

Early and late liver genes, including AFP, ALB, G-6-P, CK-18, CK-8 and CYP450-3A1, were detected in FLSPCs from both groups by semi-quantitative reverse transcription polymerase chain reaction (sQRT-PCR). Total RNA was extracted from the two FLSPC cultures (1×10^6^ cells) at days 1, 4, 7, and 14. The reverse transcription reactions were performed by hybridization at 20°C for 10 min and reverse transcription at 42°C for 12 min. The specific primers (Invitrogen, USA) for the PCR are summarized in Table 1. The PCR was performed as follows: 40 cycles of template denaturation at 95°C for 1 min, primer annealing at 65°C for 1 min and primer extension at 72°C for 2 min, followed by a final extension at 72°C for 10 min. The amplified products were separated on 3% agarose gels. The sizes of the analyzed genes were estimated by comparing the PCR products to the bands from the 50-bp DNA Ladder Marker (TAKARA, Dalian, China), which consists of 16 bands ranging from 50 bp to 1500 bp in 50-bp increments.

**Table 1:** The primers of liver genes for sQRT-PCR.

Genes	Tm (°C)	Primers(5′-3′)	Products (bp)
AFP	64.15	CAGGAGGAAGAAAGGACAAAAAA	106
	64.15	ATTCCTAAGGCATAGAAATCCCA	
ALB	63.42	GACAAAGCAGCCTGCCTGAC	174
	62.45	TTCTGCGAACTCAGCATTGG	
G-6-P	63.05	AGCCTTGTGCAACCCAGTGT	680
	62.92	AATTGCCCACCGTACACCAC	
CK-18	62.90	GCCCAGTATGAACAGCTGGC	112
	63.0	CCCTGATTTCGGCAGACTTG	
CK-8	62.91	CCGGCTTCAGCTATGGAATG	187
	63.15	GACCTCAGGCTGGCAATGAC	
CYP450-3A1	60.00	CATTCCTCACGCCAGTATATGA	198
	60.00	CGGATAGGGCTGTATGAGATTC	
GAPDH	64.15	ATGATTCTACCCACGGCAAG	89
	64.15	CTGGAAGATGGTGATGGGTT	

The stem cell markers expressed by the FLSPCs from both groups were analyzed using a FACSCalibur cytometer (Becton Dickinson, USA). After being cultured for 14 days, the cells from each group were prepared as single-cell suspensions at a density of 1×10^6^ cells/ml. These cells were separately incubated with 1 µg/ml of the CD90.1 (FITC-conjugated, Biolegend, USA) and CD49f (FITC-conjugated, Biolegend, USA) mAbs for 30 min at 4°C. All of the FLSPC samples were washed twice with wash buffer (0.1% BSA and 0.01% sodium azide) and fixed in 0.1% formaldehyde. The relevant isotype-matched mAbs, either unlabeled or labeled with the different fluorochromes, were used as negative controls.

### 2 The inductive differentiation of FLSPCs

#### 2.1 *In vitro* culture and grouping of FLSPCs

As we previously found, the optimal concentration of DMSO for induction was 1%, and the optimal concentration of HGF for induction was 20 ng/ml. Therefore, we performed the following experiments to find the most effective inductive strategy. Because the freshly amplified FLSPCs could keep their stem properties maximatily, these cells were randomly divided into four groups for the adherent culture: the control group, the DMSO-treated group (1% DMSO), the HGF-treated group (20 ng/ml HGF) and the HGF+DMSO-treated group (20 ng/ml HGF+1% DMSO). The direction of FLSPCs differentiation was mature into hepatocyte-like cells.

#### 2.2 The comparison of the secreted proteins in the four groups

The cells from each group were broken by an ultrasonic cell disruption apparatus. The supernatants were prepared for the detection of alpha-fetoprotein (AFP) and ALB expression by enzyme-linked immunosorbent assay (ELISA), which was performed strictly according to the instructions of the ELISA testing kit (GBD Corporation, San Diego, USA).

#### 2.3 The comparison of the mature liver genes in the four groups

The late liver genes (ALB, G-6-P, CK-8, CK-18 and CYP450-3A1) were detected in the induced FLSPCs from each group by sQRT-PCR. The specific primers (Invitrogen, USA) used in the PCR are described in Table 1. The protocol was clearly detailed above.

#### 2.4 The comparison of the mature liver functions

Indocyanine green (ICG) cellular uptake and elimination test was used to investigate the liver functions of metabolism. The ICG reagent (Sigma, NY, USA) was added into each 35-mm dish with medium to a final concentration of 1 mg/ml and incubated at 37°C for 15 min. The cells were then rinsed twice with PBS and incubated with fresh Williams' Medium E containing 15% FBS at 37°C. The cells were observed with an inverted microscope, and the time at which the ICG was completely pelleted was recorded as an assessment of hepatocyte function.

Glycogen synthesis test was used to evaluate the liver functions of synthesis. Cell-attached coverslips were fixed with 70% ethanol for 10 min and rinsed with deionized water. The coverslips were stained with Periodic Acid Schiff's (PAS) as per the instructions of the glycogen staining kits (Jiancheng Bioengineering Institute, Nanjing, China). The positive cells from each group were randomly counted in three high-power fields.

### 3 The identification of successful differentiation by the most effective strategy

Through the above experiments, the most effective strategy was selected and confirmed by the following experiments.

#### 3.1 The morphological comparison

The general structure and ultrastructure of the cells were observed before and after chemical induction (14 d) by a CKX-41 inverted microscope or by TEM using a JEM-1200EX microscope.

#### 3.2 The comparison of OV-6 expression

The cells before and after chemical induction (14 d) were collected and processed into single-cell suspensions. These cell suspensions were incubated with OV-6-FITC-conjugated antibodies (Bioscience, Beijing, China) (1 µg/ml) for analysis by flow cytometry.

The cell-attached coverslips from the groups before and after chemical induction (14 d) were fixed with 4% paraformaldehyde in PBS at room temperate for 15 min and were permeabilized with 0.3% Triton X-100 in PBS for 10 min. The cells were treated with 6% goat serum (Santa Cruz, CA) at room temperature for 30 min to block non-specific immune reactions and were then incubated with the anti-OV-6 primary antibody (dilution 1∶200; Santa Cruz, CA) at 4°C overnight. The cells were washed twice with PBS and incubated with the fluorescent FITC-conjugated goat anti-rabbit secondary antibody (dilution 1∶100; Santa Cruz, CA) for 2 h. Subsequently, the samples were treated with 2-(4-amidinophenyl)-6-indolecarbamidine dihydrochloride (DAPI) (dilution 1∶100; Sigma) for 15 min. The fluorescence was observed with FV1000MPE fluorescent microscope (Olympus Co, Tokyo, Japan).

### 4 The *in vitro* tracing of FLSPCs

The pAcGFP1-N1 plasmid was introduced into FLSPCs by liposome vectors. The FLSPCs (5×10^4^/ml) were seeded into 24-well plates and maintained in Williams' Medium E supplemented with 15% FBS and incubated at 37°C in a humidified atmosphere containing 5% CO_2_. When the cells covered 70% of the surface of the plate, the culture medium was replaced by serum-free medium. The pAcGFP1-N1 plasmid (5 µg) was mixed with 10 µl serum-free Williams' Medium E. A total of 10 µl of Lipofectamine (Invitrogen, California, USA) was mixed with 100 µl of serum-free Williams' Medium E. After these two components incubated for 15 min at room temperature, they were evenly mixed and incubated for an additional 45 min at room temperature to form a Lipofectamine-DNA mixture. The mixture was added into 24-well plates containing competent FLSPCs (without media). After the FLSPCs and Lipofectamine-DNA mixture were incubated for 6 h, Williams' Medium E (15% FBS) was added to the plate. Twelve hours later, the media was replaced by Williams' Medium E (15% FBS) supplemented with 500 mg/L of neomycin (Invitrogen, California, USA) for continuous culture. Thus, the uninfected FLSPCs were killed by neomycin, thereby allowing for the selection of the GFP-expressing FLSPCs. GFP expression was observed each day by inverted fluorescent microscopy, and the percentage of GFP-expressing FLSPCs was calculated.

### 5 Statistical analyses

Each analytical experiment was performed in at least triplicate (n = 3). All of the statistical analyses were performed using the SPSS 14.0 software. The data were reported as MEAN and SEM, thus t-tests were used to compare these parametric data between two groups. When there was a multiple comparison for more than two groups, ANOVA test was used. A *p*-value<0.05 was considered significant.

## Results

### 1 The *in vitro* amplification of FLSPCs

#### 1.1 The morphological comparison of the two groups of FLSPCs

**Figure 1:**
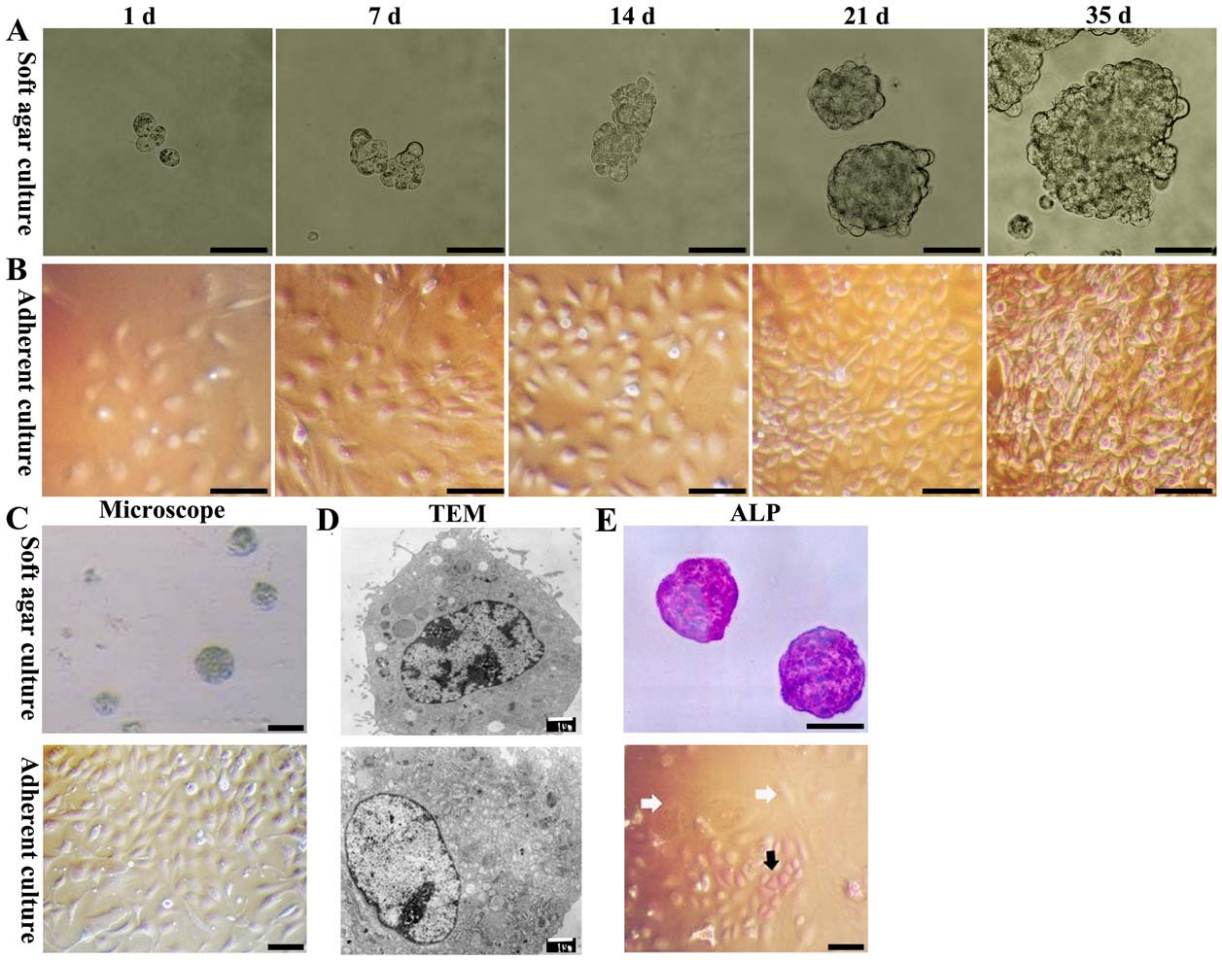
Morphological comparison of FLSPCs in adherent and soft agar cultures. (A) In the soft agar culture, the different-sized cell spheres were formed by FLSPCs at different time intervals, including days 1, 7, 14, 21 and 35. (B) In the adherent culture, FLSPCs form different cell densities at different time intervals, including days 1, 7, 14, 21 and 35. (C) Fourteen days after the initiation of cultures, FLSPCs form cell spheres containing 20–30 cells in soft agar culture. In contrast, large hepatocyte-like cells are found in the adherent culture. (D) As observed by TEM, the FLSPCs within the cell spheres have a high nuclear:cytoplasmic ratio and rich heterochromatin in the nuclei. However, some daughter cells of the FLSPCs taken from the adherent culture show a large diameter and enrichment of cytoplasmic organelles. (E) More than 90% of the FLSPCs within the cell spheres stain purple as ALP-positive cell spheres. Most of the adherent cultured FLSPCs are not stained purple (white arrows), and only 10% of the FLSPCs are stained purple (black arrow). Scale bar: 50 µm (A, B, C and E), 1 µm (D).

On the first day of culturing, the cells in the soft agar culture (Figure 1A) and the cells in the adherent culture showed no difference in proliferation (Figure 1B). Seven days later, small cell colonies containing 5–10 cells formed in the soft agar culture (Figure 1A). Meanwhile, cells in the adherent culture doubled in number and remained homogeneous (Figure 1B). After fourteen days, the cell colonies in the soft agar culture formed “mulberry-like” cell spheres containing 20–30 cells (Figure 1A), whereas the cells in the adherent culture started to form heterogeneous cell populations (Figure 1B). Twenty-one days later, homogeneous cell colonies containing 40–60 cells were generated in the soft agar culture (Figure 1A), in contrast, the cells in the adherent culture became more heterogeneous (Figure 1B). Thirty-five days after the initiation of the culture, all of the single cells that did not form cell spheres disappeared, and only cell spheres containing 80–100 cells remained in the soft agar culture (Figure 1A). Therefore, if the initial cell number was 100, then the final cell count would be 100 (initial cells)×50% (alive cells)×1 (cell spheres)×80 (cells/sphere) = 4000 cells. In other words, the cells could be amplified by 40-fold. By contrast, thirty-five days after the initiation of the culture, the cells in the adherent culture displayed a different morphology, including spindle, polygonal and other forms. Some of the large cells were hepatocyte-like cells, which contained large nuclei, lightly stained cytoplasm and prominent nucleoli (Figure 1B).

Based on the above observations, the cells in soft agar culture and adherent culture started to display significant discrepancy at 14 days. Thus, from different points of view, we compared the morphology of cells between two groups. Firstly, under light microscope, the FLSPCs in soft agar culture formed homogenous cell spheres, in contrast, the FLSPCs in the adherent culture generated heterogeneous cells (Figure 1C). Secondly, as observed by TEM, the diameters of FLSPCs in soft agar were approximately 7–13 µm. These FLSPCs had a high nuclear:cytoplasmic ratio, rich heterochromatin in the nucleus, few villous processes on the cell surface, and few organelles in the cytoplasm. All of these morphological characteristics indicate that the FLSPCs were in a naïve, undifferentiated state (Figure 1D). In contrast, the diameters of FLSPCs in the adherent culture were significantly larger (20–40 µm). These FLSPCs had a low nucleus:cytoplasm ratio, rich euchromatin within the nucleus, rich organelles in the cytoplasm, and many villous processes on their cell surfaces. These morphological characteristics reveal that FLSPCs in the adherent culture were getting close to hepatocytes. (Figure 1D). Thirdly, detected by ALP staining, more than 90% of the FLSPCs in the soft agar culture showed clear purple (Figure 1E). However, in the adherent culture, only approximately 10% of the oval FLSPCs were stained purple (Figure 1E).

#### 1.2 The functional comparison of the two groups of FLSPCs

**Figure 2:**
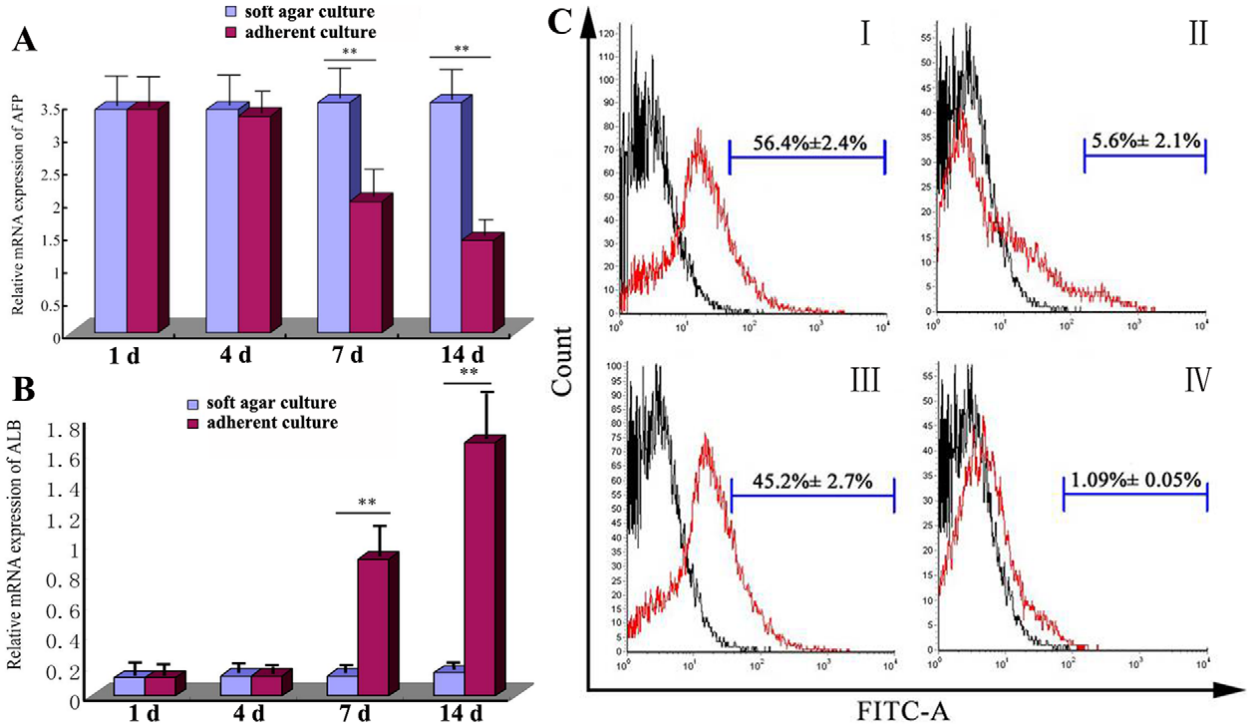
Functional comparison of FLSPCs in adherent and soft agar cultures. (A) The mRNA levels of AFP in both FLSPC cultures are displayed in a column chart. (B) The mRNA levels of ALB in both FLSPC cultures are shown in a column chart. The histograms represent the expressions of (C-I) CD90.1 in FLSPC soft agar culture, (C-II) CD90.1 in FLSPC adherent culture, (C-III) CD49f in FLSPC soft agar culture, and (C-IV) CD49f in FLSPC adherent culture.

Over time, the mRNA levels of AFP and ALB did not change significantly in any stage of the amplification of FLSPCs in the soft agar culture (Figure 2A, B) (*P*>0.05). In contrast, in the adherent culture, as time went on, the mRNA levels of AFP significantly decreased every day (Figure 2A), and the mRNA levels of ALB significantly increased each day (Figure 2B) (P<0.01).

As for expression of stem cell markers, the results were as following. Fourteen days after culture, CD90.1 was positive in more than half of the FLSPCs in the soft agar culture (56.4%±2.4%) (Figure 2 C-I), whereas only positive in 5.6%±2.1% of the FLSPCs in the adherent culture (Figure 2 C-II); CD49f was expressed in 45.2%±2.7% of the FLSPCs in the soft agar culture (Figure 2 C-III) and only expressed in 1.09%±0.05% of the FLSPCs in the adherent culture (Figure 2 C-IV). That is to say, the percentage of positive cells in soft agar culture was much higher than that in the adherent culture by10–40 multiple.

### 2 The selection of the optimal inductive strategy

#### 2.1 A comparison of the protein secretion levels for the four FLSPC groups

**Figure 3:**
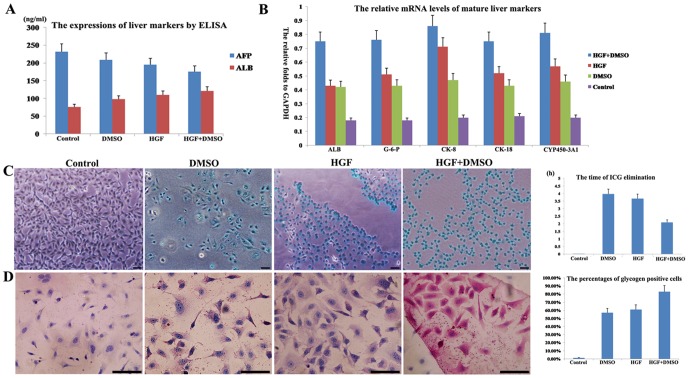
The selection of optimal inductive strategy for FLSPCs. (A) Following induction for 14 days, the expression of AFP sequentially decreases in the four groups. In contrast, the daughter cells from the FLSPCs in the control group, the DMSO-treated group, the HGF-treated group and the HGF+DMSO-treated group express increasing amounts of ALB. (B) The mRNA levels of five differentiated liver markers consistently increase in the cells from the control group, the DMSO-treated group, the HGF-treated group and the HGF+DMSO-treated group (*p*<0.05). (C) The first four pictures show the uptake of ICG by the FLSPCs in each group, and the column chart shows the time to ICG elimination in each group. (D) The first four pictures show the glycogen-granule synthesis in each group, and the column chart shows the PAS-positive cells in the four groups. Scale bar: 50 µm (C and D).

After induction for 14 days, the levels of AFP in the four groups were as follows: control, 228±21.6 ng/ml; DMSO-treated, 211±11.4 ng/ml; HGF-treated, 183±9.8 ng/ml; and HGF+DMSO-treated, 170±7.8 ng/ml. AFP expression was significantly different between any two groups (*P*<0.01). The levels of ALB expression in the four groups were as follows: control, 75±5.4 ng/ml; DMSO-treated, 100±8.3 ng/ml; HGF-treated, 112±7.8 ng/ml; and HGF+DMSO-treated, 121±10.2 ng/ml. ALB expression was also significantly different between any two groups (*P*<0.05). In summary, the daughter cells of the FLSPCs in the HGF+DMSO-treated group expressed the least AFP and the most ALB (Figure 3A).

#### 2.2 The expression of mature liver genes for the four FLSPC groups

To identify the effects of the different treatments on the targeted differentiation of FLSPCs into hepatocytes, the mRNA levels of five mature hepatic markers were detected in the four groups. The mRNA levels of five differentiated markers were consistently expressed lowest in control group, similarly moderate in HGF-treated and DMSO-treated groups, and highest in HGF+DMSO-treated group. In detail, the mRNA levels of mature liver genes (relative to GAPDH) in the HGF+DMSO-treated group were as follows: ALB (0.76±0.07), G-6-P (0.78±0.08), CK-8 (0.88±0.10), CK-18 (0.77±0.07) and CYP450-3A1 (0.81±0.08) (Figure 3B). As indicated by ANOVA tests, there were significant differences of mRNA expression between HGF+DMSO-treated group and any other group (*P*<0.05).

#### 2.3 A comparison of the mature liver functions in the four groups

The cells in the control group were not stained green upon incubation with ICG. The cells in the DMSO- and HGF-treated groups were unequally stained moderate green. In contrast, the cells in the HGF+DMSO-treated group were consistently stained a strong green (Figure 3C). After incubation with fresh medium, the dye was eliminated after 4.12±0.041 hours in the DMSO-treated group, 3.68±0.034 hours in the HGF-treated group, and 2.12±0.023 hours in the HGF+DMSO-treated group (Figure 3C).

Except in the control group, red-stained glycogen granules were observed in the cells of the DMSO-, HGF- and HGF+DMSO-treated groups (Figure 3D). The percentage of PAS-positive cells was 56.12±5.03% in the DMSO-treated group, 63.45±6.11% in the HGF-treated group, and approximately 83.26±7.85% in the HGF+DMSO-treated group (Figure 3D).

### 3 The confirmation of the optimal inductive strategy

Through our analysis, we found that the optimal inductive strategy was the combination of 20 ng/ml HGF and 1% DMSO. We identified the targeted differentiation of FLSPCs mainly by comparing the differences before and after HGF+DMSO induction (14 d). The specific results are described in the following sections.

#### 3.1 The morphological comparison

**Figure 4:**
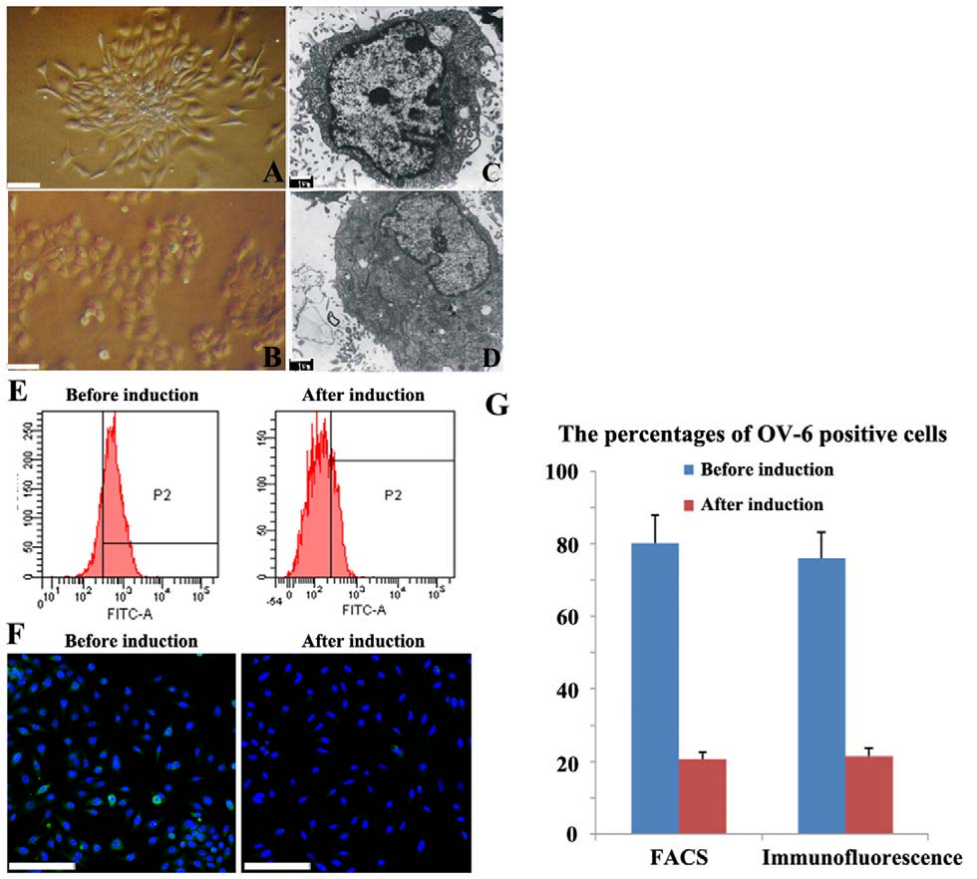
The confirmation of hepatic differentiation by HGF+DMSO induction. (A) Without induction in the adherent culture, the FLSPCs form relative homogeneous cell colonies. (B) Upon induction with HGF+DMSO, the FLSPCs generate many large cells. (C) Before induction, most of the FLSPCs have small diameters, high nuclear:cytoplasmic ratio, few organelles and few villous processes. (D) After induction, the daughter cells from FLSPCs are large, with lower nuclear:cytoplasmic ratios, more villous, and higher numbers of organelles in the cytoplasm. (E) Flow cytometry analysis shows the percentages of the OV-6 positive cells in the FLSPCs before and after induction. (F) Immunofluorescence shows the percentages of the OV-6 positive cells in the FLSPCs before and after induction. (G) The bar graphs represent the percentages of OV-6 positive cells before and after induction. Scale bar: 50 µm (A, B and F), 1 µm (C and D).

Before induction, the FLSPCs were mainly small oval cells. These small cells proliferated very rapidly and formed several cell colonies. These cell colonies appeared as concentric circles or radial shapes (Figure 4A). When the FLSPCs were induced by HGF+DMSO for 14 days, hepatocyte-like cells, which contained two or more nucleoli, were frequently found (Figure 4B).

Before induction, most of the FLSPCs had small diameters, high nuclear:cytoplasmic ratios, few organelles (including the endoplasmic reticulum, mitochondria and ribosomes), and few villous processes on the cell surfaces (Figure 4C). After induction by HGF+DMSO treatment, the differentiated cells were much larger, with much lower nuclear:cytoplasmic ratios, more organelles in the cytoplasmic compartments, and abundant villous processes on cell surfaces (Figure 4D).

#### 3.2 The comparison of OV-6 expression

By flow cytometry, before induction the percentage of OV-6-positive FLSPCs was 80.2%±7.8%, but after induction it was only 19.5%±1.7% (Figure 4E, G). By immunofluorescence, the results were similar. Before induction, 76.4%±6.5% of the FLSPCs were positive for OV-6 staining, whereas after induction by HGF+DMSO, 19.2%±1.9% of the FLSPCs were OV-6 positive (Figure 4F, G). These results indicate that the FLSPCs lost their expression of stem cell markers due to the chemical induction by HGF+DMSO.

### 4 The *in vitro* tracing of FLSPCs

The concentration of the pAcGFPI-N1 plasmid DNA was estimated to be 584 mg/L by UV spectrophotometry. The difference in the size of the DNA bands between the positively and negatively supercoiled bands was 4,700 bp, as estimated by 1% agarose gel electrophoresis. This size is corresponding to the pAcGFPI-N1 plasmid. These results indicated that the pAcGFPI-N1 plasmid was of sufficient quality and quantity for the transfection into mammalian cells.

**Figure 5:**
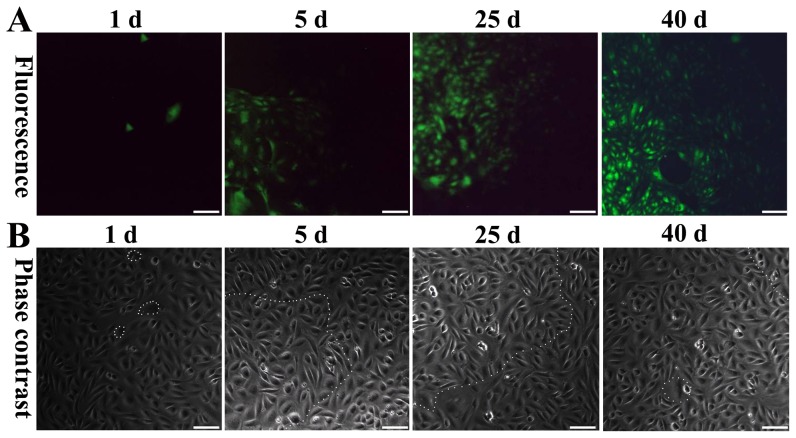
The *in vitro* tracing of FLSPCs by GFP . (A) One day after transfection, green fluorescence is observed in individual cells. Five days later, an increased number of cells express GFP and form cell colonies. Twenty-five days after the transfection, several large cell colonies, each containing dozens of FLSPCs, are found expressing GFP. Forty days after the transfection and subsequent neomycin selection, only GFP-expressing cell colonies are found. (B) The fluorescent areas are reflected by the white dotted lines in the phase contrast pictures corresponding to each fluorescent picture. Scale bar: 50 µm (A and B).

As observed by inverted fluorescence microscopy, the expression of GFP was observed in individual FLSPCs 4 h after the transfection of the pAcGFPI-N1 plasmid (Figure 5A). Five days later, a greater number of cells expressed GFP, and these cells formed colonies (Figure 5A). Following the neomycin selection 25 days after transfection, several large cell colonies containing dozens of FLSPCs were found to express GFP (Figure 5A). A total of 40 days after the transfection, only GFP-expressing cell colonies were found. The morphology of the GFP-expressing FLSPCs within the cell colonies was not noticeably different from that of the untransfected FLSPCs (Figure 5A). Phase contrast pictures corresponding to each fluorescent image are provided to illustrate the process (Figure 5B). These pictures show that although the cell density kept stable, the GFP-expressing cells gradually proliferated and took place of the unlabelled cells, which were killed by neomycin.

## Discussion

The use of stem cells and their progeny in animal models of liver disease has been encouraging and has stimulated clinical experiments [1]. In a previous study, we successfully isolated FLSPCs using a novel method [15]. To investigate their therapeutic potential, three main issues need to be solved: 1) *in vitro* culturing to guarantee the efficient self-renewal of FLSPCs, 2) *in vitro* induction to push FLSPCs differentiation into functional mature cells, including hepatocytes; and 3) *in vitro* labeling to easily and steadily monitor FLSPCs. At present, most of the investigations only concentrate on one aspect of the above three problems [30]. Meanwhile, the present strategies are not effective enough. In this study, we made three important improvements to cover all the above mentioned problems. Briefly, 1) based on a novel *in vitro* soft agar culture technique, FLSPCs could be amplified very efficiently and maintained in an undifferentiated state; 2) through a highly efficient inductive strategy (HGF+DMSO treatment), FLSPCs were capable of producing hepatocyte-like cells; 3) by GFP labeling, FLSPCs could be steadily and easily traced *in vitro*. These findings will contribute to stem cell-based therapies to treat liver diseases, at least in animal models.

The primary limitation of the use of FLSPCs in therapeutic applications is the necessity for a large number of undifferentiated cells [31]. As a result, a high-yield culture protocol is urgently needed. In a previous study, we found that many FLSPCs died in adherent culture due to the lack of attachment or overt cell differentiation. What's more, repeated passaging using the adherent culture reduces the pluripotency and the proliferation capacity of stem cells [32]. Thus, it was urgent to find another effective culture method. Advances made in the techniques for the large-scale culture of other stem cells will facilitate the expansion of undifferentiated FLSPCs. For large and rapid expansion of NSCs, spheroid inoculum forms are preferred [33]. This culture method may be well suited to analyze the plasticity, cell-cell, and cell-extracellular matrix (ECM) interactions of stem cells [16], [32]. In addition to culture method, to maintain stem cells in an undifferentiated state, feeder cells or differentiation inhibitory factors (DIF) are needed [34]; however, using feeder cells in stem cell cultures is highly laborious and limits large-scale stem cell production for potential applications in regenerative medicine [35]. Fortunately, the proper DIF can perform the role of feeder cells [36]. Among DIFs, LIF is one of the most effective factor for maintaining the pluripotency of stem cells [37]. Here, we describe a new soft agar culture technique with LIF to scale-up the production of FLSPCs in undifferentiated state. In contrast, in the adherent culture with LIF, many FLSPCs differentiated into hepatocyte-like cells. We speculate that the differences in proliferation, apoptosis, and differentiation of FLSPCs may be mainly due to the different culture method. However, in this study, FLSPCs were cultured in a static state, which might be not as effective as a dynamic state to retain the full multipotent characteristics of stem cells [32], [38], [39].

The inductive conditions for the hepatic differentiation of FLSPCs remain unclear. Investigating the traits of hepatic differentiation in FLSPCs will be helpful because it is the key to realizing the therapeutic potential of FLSPCs. So far, the molecules known to be able to induce differentiation include interferon-alpha, interleukin-4, tumor necrosis factor-alpha (TNF-alpha), HGF and DMSO [21]. Since the initial cloning and characterization of HGF as a mitogen for hepatocytes [40], increasing studies have revealed that hepatic differentiation and maturation of cells occurs upon exposure to HGF [18], [19], [41], [42]. In addition to HGF, DMSO can be used to induce hepatic differentiation [20]. However, when separately used, the effects of hepatic induction by these two molecules are not satisfying. It is not clear whether combing HGF with DMSO, the FLSPCs could be better induced to differentiate into hepatocytes. To prove the above hypothesis, in this study, by exposure to HGF, DMSO, or HGF+DMSO, we tried to induce the differentiation of FLSPCs. Successful hepatic differentiation could be identified by the morphological observation of hepatocytes, the expression of hepatocyte-specific markers (ALB, G-6-P, CK-8, CK-18 and CYP450-3A1), and the detection of synthetic and metabolic liver functions [43]. According to the above criteria, we found that the combination of HGF and DMSO was the most effective strategy to induce hepatic differentiation of FLSPCs.

The generation of labeled FLSPCs is essential to confirm the therapeutic effect of FLSPCs when they are used in the treatment of liver diseases. In particular, this labeling will facilitate the real-time monitoring of differentiation into hepatocyte cells both *in vitro* and *in vivo* [22], [44]. To address these questions, a plasmid containing the GFP gene was transfected into FLSPCs. As a result, the GFP-labeled FLSPCs were readily visualized by their intense green fluorescence. In this way, the progeny of differentiated FLSPCs could be analyzed and sorted based on their GFP expression.

To conclude, in this study we have made three major findings concentrating on FLSPCs based cell therapy. Firstly, we used a new soft agar culture with LIF to keep FLSPCs in undifferentiated state when amplification. Secondly, we combined HGF and DMSO together to induce FLSPCs differentiation, which was more effectively than the conventional strategies. Thirdly, with GFP label, FLSPCs were readily traced for later potential investigation. These findings can help to ensure that FLSPCs are in the proper status for cell therapy, so that it will facilitate promoting a thorough investigation of FLSPCs and their therapeutic potential.

## Conclusions

Before the therapeutic potential of FLSPCs can be investigated, many challenges, including the maintenance of the undifferentiated state, the efficiency of inducing cell differentiation, and the stability of tracing cells, must be overcome. This study successfully conquered the above challenges by making three important *in vitro* improvements: firstly, soft agar culture could guarantee FLSPCs self-renewal in large-scale; secondly, HGF+DMSO treatment could effectively induce FLSPCs differentiation into hepatocytes; thirdly, with GFP label, FLSPCs could be reliably traced. The insights gained from this study will be helpful in designing optimal strategies for treating liver diseases based on FLSPCs.

### Limitations

Our study has two main limitations. Firstly, although we have made some improvements, the strategy is not optimized. For example, whether a dynamic culture is more suitable than a static culture for FLSPCs needs to be verified. Secondly, although we previously demonstrated that the transplantation of FLSPCs induces the regeneration of injured livers in adult rats [14], the exact role of the participation of FLSPCs in liver regeneration and its mechanisms need to be thoroughly investigated.
