# Correction: Several Important *In Vitro* Improvements in the Amplification, Differentiation and Tracing of Fetal Liver Stem/Progenitor Cells

**DOI:** 10.1371/annotation/1bd1a50d-b991-4210-a5d2-78a588e01d1e

**Published:** 2013-05-10

**Authors:** Wei-hui Liu, Zheng-cai Liu, Nan You, Ning Zhang, Tao Wang, Zhen-bin Gong, Hong-bao Liu, Ke-feng Dou

There was an error in the Funding statement.

The correct version of the Funding is: This study was supported by the National Natural Science Foundation of China (No. 81170419, 81172061, 81000309) and Nature Science Foundation of Shaanxi Province (No. 2007K09-05(7)). The funders had no role in study design, data collection and analysis, decision to publish, or preparation of the manuscript. 

